# Does emotion dysregulation mediate the association between ADHD symptoms and internalizing problems? A longitudinal within‐person analysis in a large population‐representative study

**DOI:** 10.1111/jcpp.13624

**Published:** 2022-04-29

**Authors:** Evelyn Mary‐Ann Antony, Milla Pihlajamäki, Lydia Gabriela Speyer, Aja Louise Murray

**Affiliations:** ^1^ Department of Psychology University of Edinburgh Edinburgh UK

**Keywords:** Attention‐deficit/hyperactivity disorder, emotion dysregulation, internalizing problems, child development, autoregressive latent trajectory model with structured residuals

## Abstract

**Background:**

Previous research has suggested that children with attention‐deficit/hyperactivity disorder (ADHD) symptoms commonly show emotion dysregulation difficulties. These difficulties may partly explain the strong tendency for internalizing problems such as anxiety and depression to co‐occur with ADHD symptoms. However, no study has yet provided a longitudinal analysis of the within‐person links between ADHD symptoms, emotion dysregulation, and internalizing problems necessary to examine this hypothesis from a developmental perspective.

**Methods:**

We used data from the age 3, 5, and 7 waves of the large UK population‐representative Millennium Cohort Study (*n* = 9,619, 4,885 males) and fit gender‐stratified autoregressive latent trajectory models with structured residuals (ALT‐SR) to disaggregate within‐ and between‐person relations between ADHD symptom, emotion dysregulation, and internalizing problem symptoms.

**Results:**

We found that emotion dysregulation significantly mediated the longitudinal within‐person association between ADHD symptoms and internalizing problems.

**Conclusions:**

Results underline the promise of targeting emotion dysregulation as a means of preventing internalizing problems co‐occurring with ADHD symptoms.

## Introduction

Attention‐deficit/hyperactivity disorder (ADHD) is characterized by significant difficulties in the domains of attention and/or hyperactivity/impulsivity (American Psychiatric Association [APA], 2013). It is estimated that approximately 5% of children meet diagnostic criteria for the condition; however, it is also widely acknowledged that symptoms are on a continuum, meaning that many more children are affected at subclinical levels (Balázs & Keresztény, 2014). Emotion dysregulation difficulties are commonly associated with ADHD symptoms, and have been proposed as a potential mediating mechanism by which ADHD symptoms engender elevated risk for issues that commonly co‐occur with ADHD symptoms, including internalizing problems such as anxiety and depression (Anastopoulos et al., 2011; Murray, Wong, et al., 2020; Rosen & Factor, 2015). However, research on the role of emotion dysregulation in co‐occurring issues with ADHD to date has been limited to analyses that do not disentangle within‐ and between‐person relations. The goal of the current study was thus to evaluate the mediating role of emotion dysregulation in ADHD‐internalizing symptom co‐occurrence by applying the autoregressive latent trajectory model with structured residuals (ALT‐SR) to a large longitudinal population‐representative study.

Emotion dysregulation has been characterized as the expression and experience of emotions which are excessive relative to social norms, context, and developmental stage; that involve rapid and poorly controlled changes in emotion; and that involve anomalous allocation of attention to emotional stimuli (Shaw, Stringaris, Nigg, & Leibenluft, 2016). Thus defined, emotion dysregulation is prevalent among individuals with ADHD symptoms, estimated to affect 25–45% of children with ADHD (Shaw, Stringaris, Nigg, & Leibenluft, 2014). Indeed, its prevalence is such that it is debated as a potential core feature to be added in future diagnostic criteria for ADHD (Faraone et al., 2019; Nigg et al., 2020; Posner, Kass, & Hulvershorn, 2014).

Emotion regulation difficulties are also related to anxiety and depression, and have been proposed to play a role in the elevated rates of these issues among children with ADHD symptoms. From a developmental cascades perspective, for example, emotion regulation issues have been proposed to act as a bridge between ADHD symptoms and other domains of mental health, thus being partially responsible for the accumulation of further issues such as anxiety and depression over development (Steinberg & Drabick, 2015). Evidence suggests that 15–75% of youth with ADHD meet diagnostic criteria for depression and 25% meet diagnostic criteria for an anxiety disorder (Biederman, Newcorn, & Sprich, 1991; Jarrett & Ollendick, 2008; Kim et al., 2009; Krone & Newcorn, 2015; Schatz & Rostain, 2006). The association extends into the nonclinical range, reflecting that both ADHD symptoms and internalizing problems sit on a continuum (Balázs & Keresztény, 2014; Murray, Caye, et al., 2022).

Relatively few studies have, however, examined the role of emotion dysregulation in the co‐occurrence of internalizing problems with ADHD symptoms in children. Anastopoulos et al. (2011) found that emotion dysregulation mediated the relations between ADHD and internalizing problems in a cross‐sectional study of children aged 5–12. Further, using ecological momentary assessment (EMA) to capture emotion dysregulation, Rosen and Factor (2015) found that emotion dysregulation was associated with internalizing problems in a sample of *n* = 27 children with ADHD. Using a similar design, Leaberry, Rosen, Fogleman, Walerius, and Slaughter (2017) found that children with ADHD and a co‐occurring internalizing disorder showed higher levels of emotion dysregulation. One study, however, found no association between ADHD diagnostic status and EMA‐derived emotion dysregulation in a sample of *n* = 102 children, *n* = 56 of whom had ADHD, though they did find an association between emotion dysregulation and internalizing problems (Rosen, Walerius, Fogleman, & Factor, 2015).

Though pre‐existing evidence is, on balance, consistent with the idea that emotion dysregulation plays a role in the increased risk of internalizing problems associated with ADHD symptoms, there remains a question over what the associations identified to date reflect. Recent discussions have highlighted that despite many theories in developmental psychopathology referring to processes occurring at the within‐person level, the modeling techniques most commonly used actually yield parameters that reflect a mixture of within‐ and between person effects (Berry & Willoughby, 2017; Hamaker, Kuiper, & Grasman, 2015; Mund & Nestler, 2019). Here the between‐person effects—reflecting relatively stable individual differences—can differ in magnitude and direction from the within‐person effects and thus confuse attempts to estimate the latter (Berry & Willoughby, 2017; Murray, Eisner, & Ribeaud, 2020). Within‐person effects are also typically of the greatest significance for informing interventions that directly target the constructs in a hypothesized pathway (Hamaker et al., 2015). For example, interventions targeting emotion dysregulation problems would be promising only if an individual with ADHD symptoms who successfully increases their emotion regulation abilities would be expected to show a decrease in their internalizing problems. If, however, associations between ADHD symptoms, emotion dysregulation, and internalizing problems reflect a common dependence on stable individual differences between people (e.g., due to time‐stable shared genetic/temperamental effects), then such interventions may be targeting the wrong factors.

Models such as the autoregressive latent trajectory model with structured residuals (ALT‐SR) are helpful for disaggregating within‐ and between‐person effects and are thus growing in popularity within developmental psychopathology (Curran, Howard, Bainter, Lane, & McGinley, 2014). The ALT‐SR is based on fitting a cross‐lagged structure to the residuals of a parallel process growth curve model where the latter part models between‐person differences, allowing the within‐person effects to be estimated free from confounding by stable individual differences (Curran et al., 2014). The model can also be extended to test mediation (Murray, Obsuth, et al., 2021).

Finally, it is important to consider gender differences in the role of emotion dysregulation in linking ADHD symptoms with internalizing problems. Evidence suggests that while ADHD symptoms are more common in males, internalizing problems are more common in females (and more commonly co‐occur with ADHD symptoms in females) (Gershon & Gershon, 2002; Steinberg & Drabick, 2015). Taken together with the gender differences in emotion regulation, it has been argued that more research on gender differences in ADHD, emotion regulation, and links with co‐occurring issues is warranted (Steinberg & Drabick, 2015). Reviewing the evidence on the role of emotion regulation in ADHD and co‐occurring mental health issues, for example, Steinberg and Drabick (2015) discussed a set of hypotheses that implied a stronger mediating role for emotion regulation difficulties in girls compared with boys due to an increased risk of these difficulties manifesting in depression in the former compared with the latter.

In the current study we, therefore, fit ALT‐SR models stratified by gender to three waves of data from the UK Millennium Cohort Study (Connelly & Platt, 2014) to provide the first within‐person analysis of whether emotion dysregulation mediates the links between ADHD symptoms and internalizing problems in childhood. Given that issues such as problems with peers, parents, and school have previously been identified as mediators of cascades between ADHD symptoms and internalizing problems (Humphreys et al., 2013; Roy, Hartman, Veenstra, & Oldehinkel, 2015), we hypothesized that emotion dysregulation problems would be a partial but significant mediator of the relation between ADHD symptoms and internalizing problems in childhood.

## Methods

### Ethics

The Millennium Cohort Study (MCS) received full ethical approval from the National Health Service Multi‐Centre Research Ethics Committee at each wave (Connelly & Platt, 2014).

### Participants

Millennium Cohort Study is a longitudinal study of a representative sample of children born into 19,244 families in the United Kingdom between 1 September 2000 and 31 August 2001 for England and Wales, and 24 November 2000 and 11 January 2002 for Scotland and Northern Ireland (Plewis, 2007). The initial sample included all children born during these timeframes who were living in the United Kingdom at 9 months of age, and eligible to receive Child Benefit (Plewis, 2007). A stratified design and weights provided by the MCS were used to ensure adequate representation of disadvantaged and ethnic minority children and to handle nonrandom attrition (Plewis, 2007). Fuller descriptions of the design and procedure can be found elsewhere (Hansen, 2014; Plewis, 2007). In this study, complete case data from wave 2 at 3 years of age, wave 3 at 5 years of age, and wave 4 at 7 years of age were used, giving *n* = 9619 (*n* = 4734 females), who participated up until wave 4.

#### Child Social Behaviour Questionnaire

Emotion dysregulation was measured using the *Emotional Dysregulation* subscale of *Child Social Behaviour Questionnaire* (CSBQ; Hartman, Luteijn, Serra, & Minderaa, 2006). It contains five items (e.g., “gets over excited”) administered in the self‐completion module of the parent‐interview at ages 3, 5, and 7. Parents were asked to think about their child’s behavior during the past 6 months, and to choose whether each statement was: Not true (1), Somewhat true (2), Certainly true (3), or Can’t say (4). The latter category was treated as missing in the analyses. Sum scores were calculated after reverse coding as relevant (range 5–15). The items are provided in the Supporting Information, Appendix S1. The psychometric properties of the CSBQ scores have been examined in previous studies, supporting their reliability, structural validity, and criterion validity (Hartman et al., 2006). In the current sample, omega reliability (McDonald, 1999) was good at .72 for age 3, .77 for age 5, and .79 for age 7. Omega was used as it provides a better reliability estimate than Cronbach’s alpha (Peters, 2018).

#### Strengths and Difficulties Questionnaire

ADHD symptoms and internalizing problems were measured using the *Strengths and Difficulties Questionnaire* (SDQ; Goodman, 1997). Internalizing problems were measured using the Emotional Symptoms scale, which contains five items related to emotional problems (for example, “often seems worried”; Goodman, 1997, 2001). The same five items were used at each wave. ADHD symptoms were measured using the Hyperactivity/Inattention scale, which contains five items related to the key symptom domains of ADHD (APA, 2013; Goodman, 1997, 2001). In line with the recommended administration of the SDQ, the item “can stop and think before acting” was used at age 3 instead of “thinks things out before acting” to ensure its age‐appropriateness.

During the self‐completion module of the parent interview at ages 3, 5, and 7, parents were asked to think about their child’s behavior during the past 6 months. Responses were recorded on a three‐point scale from “Not true” (0) to “Certainly true” (2). Respondents could also select a “Can’t say” option, which was treated as missing in the analyses. Sum scores were calculated after reverse scoring items where relevant (range 0–10). Paraphrases of item contents are provided in Supporting Information.

The psychometric properties the SDQ have been extensively examined (see Kersten et al., 2016 for a review), including in the current sample (Murray et al., 2021a, 2021b). These studies have mostly confirmed the reliability and validity of its scores (Goodman, 1997, 2001; Goodman, Meltzer, & Bailey, 1998). In the current sample, omega reliability (McDonald, 1999) was good at .74 for age 3, .76 for age 5, and .79 for age 7 for the Emotional Symptoms scale, and at .75 for age 3, .82 for age 5, and .76 for age 7 for the Hyperactivity/Inattention scale.

### Statistical procedure

Autoregressive latent trajectory models with structured residuals (Curran et al., 2014) were fit in R (version 3.6.1; R Core Team, 2019), using the *lavaan* (version 0.6.7; Rosseel, 2012) and *lavaan.survey* packages (version 1.1.3.1; Oberski, 2014) to account for the complex survey design. The models are described comprehensively in previous publications (Berry & Willoughby, 2017; Curran et al., 2014; Mund & Nestler, 2019; Speyer et al., 2020). In brief, the ALT‐SR is based on fitting a cross‐lagged structure to the residual factors from a parallel process growth curve model with random intercepts and fixed slopes. Between‐person associations are captured in the covariance between the random intercept factors. In addition, within‐person associations are captured in the autoregressive, cross‐lagged, and concurrent (residual) covariance parameters of the cross‐lagged structure fit to the growth curve residuals. This allows time‐stable between‐person effects to be partialed out of the within‐person effects. Mediation was evaluated by calculating the product of the coefficients for the within‐person effects of ADHD symptoms on emotion dysregulation and of emotion dysregulation on internalizing problems. Direct effects of ADHD symptoms in internalizing problems were also included in the model. The significance of the indirect effect parameter was assessed in two ways: (a) using the delta method combined with a robust maximum likelihood estimator that accounted for the complex sampling design of the MCS, and (b) using bootstrapping combined with the standard maximum likelihood estimator. Both methods were used because the delta method may be too conservative (it incorrectly assumes a symmetric sampling distribution of the indirect effects), but it is not currently straightforward to combine complex survey design estimators with bootstrapping.

Attrition weights provided by the MCS were used to handle nonrandom attrition (Murray, Ushakova, Wright, Booth, & Lynn, 2021). This means that only the participants who responded up until the wave 3 are included in the analyses as those who did not are assigned a weight of 0. Attrition weights deal with attrition bias introduced due to nonrandom drop‐out by up‐weighting cases with a low probability of response and down‐weighting those with a high probability of response. They provide unbiased parameter estimates under a “missing at random” (MAR) assumption, that is, that the missingness is random conditional on the (auxiliary) variables modeled. Analyses were stratified by gender to allow for gender differences in the relations between ADHD symptoms, emotion dysregulation, and internalizing problems. The model was also fit to the whole sample without stratification. A sensitivity analysis was conducted omitting the “impulsivity” item of the emotion dysregulation scale to ensure that mediating effects were not driven by an overlap in content between the ADHD and emotion dysregulation measures.

Models were unadjusted for covariates because the fact that in the ALT‐SR each individual is compared to themselves over time means that it automatically adjusts for unmeasured time‐stable between‐person effects (Speyer, Hall, Hang, Hughes, & Murray, 2021). This may include, for example, the potential confounding effects of gender, genetics, and stable aspects of the family environment. It is, however, vulnerable to the effects of unmeasured time‐varying confounds (e.g., an event affecting both emotional problems and emotional regulation at a particular measurement wave).

## Results

**Figure 1:**
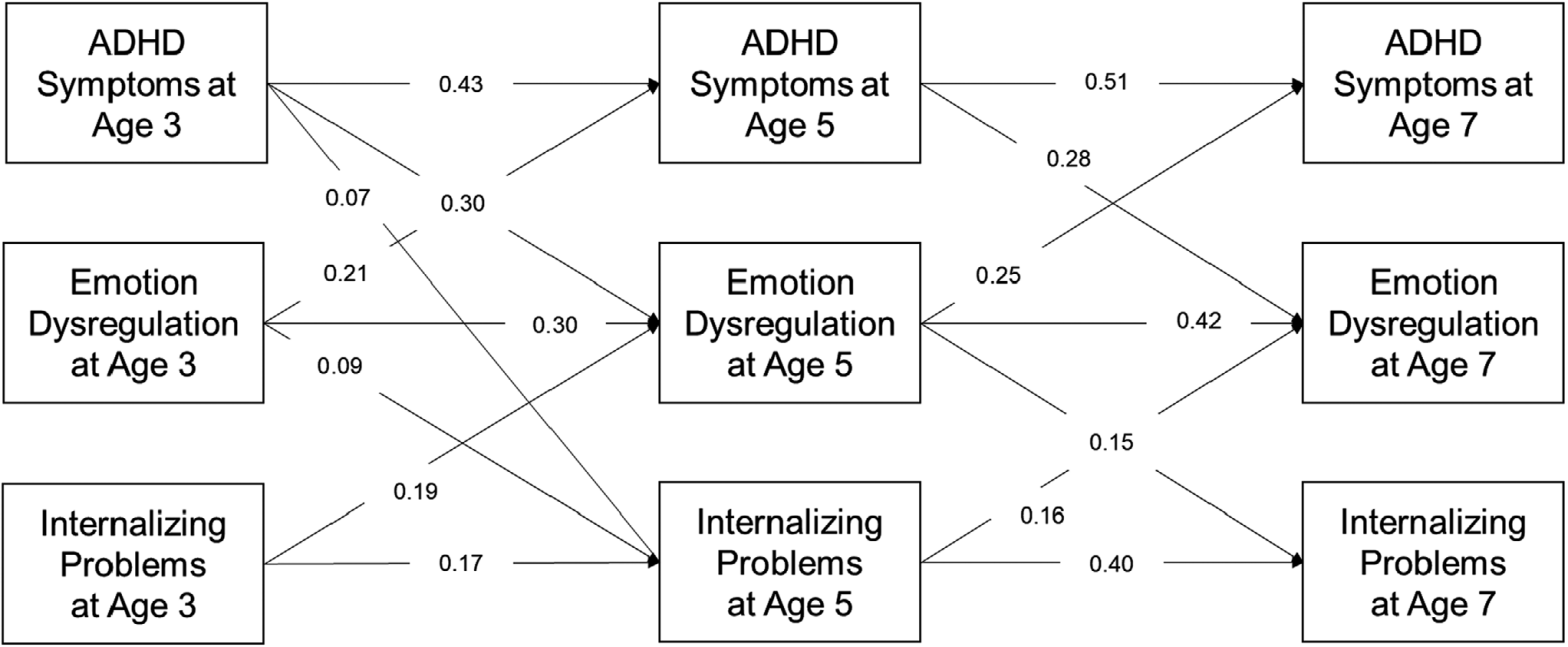
Unstandardized autoregressive and cross‐lagged estimates from the ALT‐SR fit to the male subsample. *Note*. Solid lines represent statistically significant paths at *p* < .05. Latent growth curve and covariance parameters are omitted for clarity

**Figure 2:**
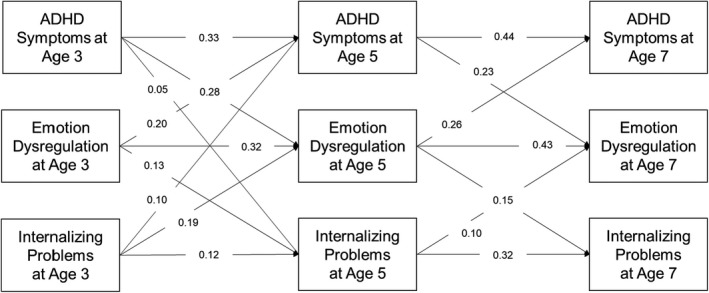
Unstandardized autoregressive and cross‐lagged estimates from the ALT‐SR fit to the female subsample. *Note*. Solid lines represent statistically significant paths at *p* < .05. Latent growth curve and covariance parameters are omitted for clarity

Descriptive statistics are provided in Table 1. The cross‐lagged parameters from the ALT‐SR models are provided in Figures 1 and 2. As noted above, two methods were used: a design‐adjusted estimation with the delta method to assess the significance of indirect effects, and an ML‐estimated model using bootstrapping to assess the significance of indirect effects. The latter was a sensitivity check given that the delta method has limitations in representing the true sampling distribution of indirect effects (MacKinnon, Lockwood, Hoffman, West, & Sheets, 2002; MacKinnon, Lockwood, & Williams, 2004). The sensitivity analysis omitting the “impulsivity” item yielded the same pattern of results, the main difference being that the effect of age 3 emotion dysregulation on age 5 ADHD symptoms was smaller (but still significant) when omitting the “impulsivity” item. As the results from these different methods were highly similar, only the design‐adjusted results using the full emotion dysregulation scores are provided below with full results for sensitivity analyses available at: https://doi.org/10.17605/OSF.IO/XGHMY. Model results for the whole sample (not stratifying by gender) are also provided at this link. These were similar to those in the gender‐stratified models. Standardized parameter estimates for males and females are provided in Supporting Information (Figures S1 and S2).

**Table 1:** Descriptive statistics at ages 3, 5, and 7

	*N*	*M*	*SD*	Min	Max	Range
CSBQ Emotional Dysregulation (Age 3)	9,619	9.33	2.27	5	15	10
SDQ Emotional Symptoms (Age 3)	9,619	1.30	1.41	0	10	10
SDQ Hyperactivity/Inattention (Age 3)	9,619	3.82	2.36	0	10	10
CSBQ Emotional Dysregulation (Age 5)	9,619	8.51	2.30	5	15	10
SDQ Emotional Symptoms (Age 5)	9,619	1.31	1.53	0	10	10
SDQ Hyperactivity/Inattention (Age 5)	9,619	3.20	2.37	0	10	10
CSBQ Emotional Dysregulation (Age 7)	9,619	8.49	2.38	5	15	10
SDQ Emotional Symptoms (Age 7)	9,619	1.48	1.72	0	10	10
SDQ Hyperactivity/Inattention (Age 7)	9,619	3.28	2.53	0	10	10

### Males

Unstandardized parameter estimates are provided in Figure 1. On balance, the hypothesized model was judged to satisfactorily fit the data (CFI = 0.97, TLI = 0.86, RMSEA = 0.13, SRMR = 0.04). The poor TLI value likely reflects the lack of parsimony of the model; however, the proper specification of the ALT‐SR requires the inclusion of a large number of parameters. Examining modification indices and expected parameter changes for the model suggested that either freeing some intercept and slope factor loadings or freely estimating the slope factor variance could improve model fit. However, given that CFI and SRMR suggested good fit, these changes were not implemented because they would fundamentally alter the interpretation of the model (see e.g., Speyer et al., 2020 for a discussion). The indirect effect of ADHD symptoms on internalizing problems via emotion dysregulation was significant (*b* = 0.04, 95% CI = 0.03–0.06, *p* < .001) but the direct effect was not (*b* = −0.02, 95% CI = −0.04–0.01, *p* = .27).

### Females

Unstandardized parameter estimates are provided in Figure 2. On balance, the model was judged to satisfactorily fit the data (CFI = 0.97, TLI = 0.84, RMSEA = 0.13, SRMR = 0.04). Examining modification indices and expected parameter changes suggested similar changes as for the male sub‐sample; however, again, these changes were not implemented because they would fundamentally alter the interpretation of the model. The indirect effect of ADHD symptoms on internalizing problems via emotion dysregulation was statistically significant (*b* = 0.04, 95% CI = 0.03–0.06, *p* < .001) but direct effect was not (*b* = 0.01, 95% CI = −0.02–0.04, *p* = .61).

## Discussion

Internalizing problems often co‐occur with ADHD symptoms (Jarrett & Ollendick, 2008) and emotion dysregulation has been hypothesized as a key linking mechanism. Though preliminary evidence has been consistent with this hypothesis (Anastopoulos et al., 2011; Murray, Wong, et al., 2020; Rosen & Factor, 2015), our study is the first to provide the critical longitudinal, within‐person analysis needed to robustly test this claim. Our findings of significant mediating effects in both males and females thus provide some of the most compelling evidence to date that associated emotion dysregulation problems may play a critical role in the development of internalizing problems among children with elevated ADHD symptoms. The effect sizes were relatively small; however, as has been argued in detail elsewhere (Adachi & Willoughby, 2015), in a longitudinal modeling context these can still represent important effects.

Our results suggest that, consistent with a developmental cascades model (Masten & Cicchetti, 2010), emotion regulation issues are a mediator in the developmental links between ADHD symptoms and internalizing problems. This adds to past evidence that has identified factors such as peer problems, problems with parents, and academic issues as links in this chain over the course of development (Humphreys et al., 2013; Powell et al., 2020; Roy et al., 2015). It can thus contribute to the further development of cascade models that recognize the range of individual and social factors that play a role in the development of internalizing problems among children with elevated ADHD symptoms.

There are a number of potential clinical implications of our findings. Our findings confirm the importance of emotion regulation issues in relation to ADHD, and can thus inform debates about the role of emotion dysregulation in diagnostic criteria for ADHD (Faraone et al., 2019). For example, while emotion regulation issues are not unique to ADHD, their prevalence and impacts in this group suggest there may be benefits to screening for emotion dysregulation among children who meet diagnostic criteria for ADHD. This could be helpful for targeting preventive interventions to minimize the accumulation of additional internalizing problems. Findings also suggest that emotion dysregulation itself may be a key target for intervention among children with elevated ADHD symptoms. While there is some evidence that emotion dysregulation improves with standard treatments for ADHD, the effects are smaller than those for ‘core’ ADHD symptoms and only in the small‐to‐moderate range (Lenzi, Cortese, Harris, & Masi, 2018; Posner et al., 2014). This suggests that dedicated interventions for reducing and managing emotion dysregulation (e.g., Loevaas et al., 2019) could be helpful for many children with ADHD. These could draw, for example, on approaches such as mindfulness, which have shown some preliminary evidence of effectiveness for improving emotion regulation in ADHD (Huguet, Eguren, Miguel‐Ruiz, Vallés, & Alda, 2019). However, when applying existing approaches for targeting emotion dysregulation to populations of youth with elevated ADHD symptoms, care must be taken to ensure they are suitably adapted. Children with ADHD, for example, may not be able to engage as easily with long sessions which are insufficiently stimulating, or that require high levels of self‐regulation and freedom from distraction (Evans et al., 2018; Lo et al., 2020; Van der Oord, Bögels, & Peijnenburg, 2012).

### Limitations and future directions

A primary limitation of the current study was that parents were the only informants, whereas a multi‐informant perspective would be important for capturing symptoms in different settings, and in interaction with different adults (Murray, Booth, Ribeaud, & Eisner, 2018). Second, emotion dysregulation was measured with a unidimensional, retrospective measure that does not reflect all of the broad set of processes such as the awareness, understanding, acceptance, and modulation of emotions (Gratz & Roemer, 2004). Similarly, only brief measures of ADHD and internalizing symptoms were available; therefore, differential effects related to subdimensions of ADHD or internalizing symptoms could not be examined. Third, we considered the role of emotion regulation issues in only one commonly co‐occurring issue related to ADHD symptoms. However, emotion regulation has been proposed to play a role in a wide range of issues known to commonly occur with ADHD symptoms, including oppositional defiant and conduct problems, peer problems, and substance use later in life. Research that examines these links in longitudinal studies, especially over a longer period of development would also be valuable. Similarly, emotion dysregulation is increasingly recognized as a trans‐diagnostic factor associated with a wide range of mental health issues. Therefore, it would be valuable for future research to examine its role as both a common cause of, and mediator of the links between, multiple mental health issues.

Finally, we used a community ascertained sample. While this has the advantage of providing a picture of how ADHD symptoms, emotion regulation, and internalizing problems are related in the general population, it would be valuable to extend this work into samples of children with a clinical diagnosis of ADHD. It is possible that these relations differ in clinical versus nonclinical populations.

## Conclusion

Emotion dysregulation mediates the association between ADHD symptoms and internalizing problems in childhood, suggesting that it is an important target for intervention for preventing important co‐occurring issues in ADHD.

## Supporting information


**Appendix S1**. Questionnaire items.
**Figure S1**. Standardized autoregressive and cross‐lagged estimates from the ALT‐SR fit to the male subsample.
**Figure S2**. Standardized autoregressive and cross‐lagged estimates from the ALT‐SR fit to the female subsample.
