# Supplementary material for: Does emotion dysregulation mediate the association between ADHD symptoms and internalizing problems? A longitudinal within‐person analysis in a large population‐representative study
Source: J Child Psychol Psychiatry. 2022 Apr 29;63(12):1583–90. doi: 10.1111/jcpp.13624 (PMC9790420; doi:10.1111/jcpp.13624)
Supplement: Supplementary file 1 — Appendix S1. Questionnaire items. Figure S1. Standardized autoregressive and cross‐lagged estimates from the ALT‐SR fit to the male subsample. Figure S2. Standardized autoregressive and cross‐lagged estimates from the ALT‐SR fit to the female subsample. [file JCPP-63-1583-s001.docx]

**Supporting Information**

**Appendix S1. Questionnaire Items**

CSBQ Emotional Dysregulation scale

Shows mood swings

Gets over excited

Easily frustrated

Gets over being upset quickly*

Acts impulsively

SDQ Emotional Symptoms scale

Complains of headaches/stomach-aches/sickness

Often seems worried

Often unhappy

Nervous or clingy in new situations

Many fears, easily scared

SDQ Hyperactivity/Inattention scale

Restless, overactive, cannot stay still long

Constantly fidgeting

Easily distracted

Thinks things out before acting (ages 5, 7)*; Can stop and think before acting (age 3)*

Seeks tasks through to the end*

**Figures**

**
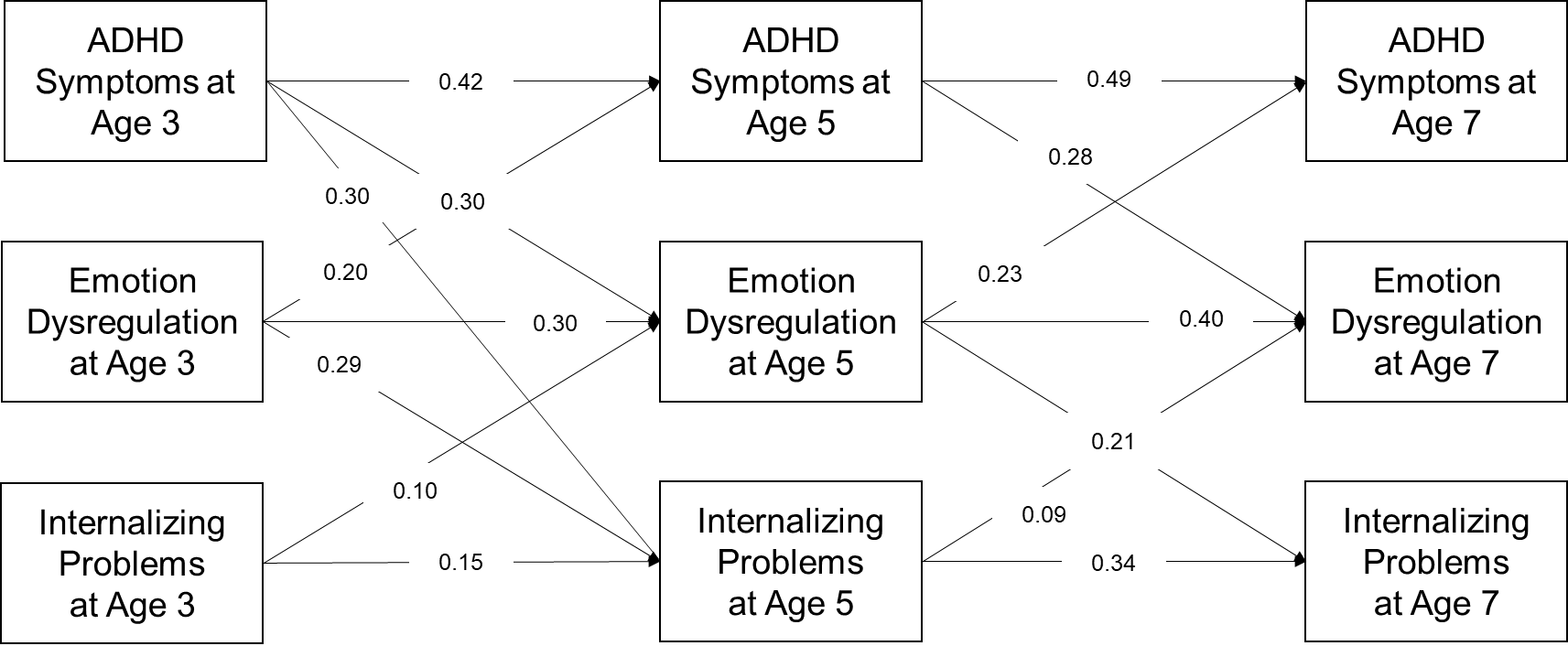
**

**Figure S1: Standardized autoregressive and cross-lagged estimates from the ALT-SR fit to the male subsample**

*Note.* Solid lines represent statistically significant paths at *p* <.05. Latent growth curve and covariance parameters are omitted for clarity.

**
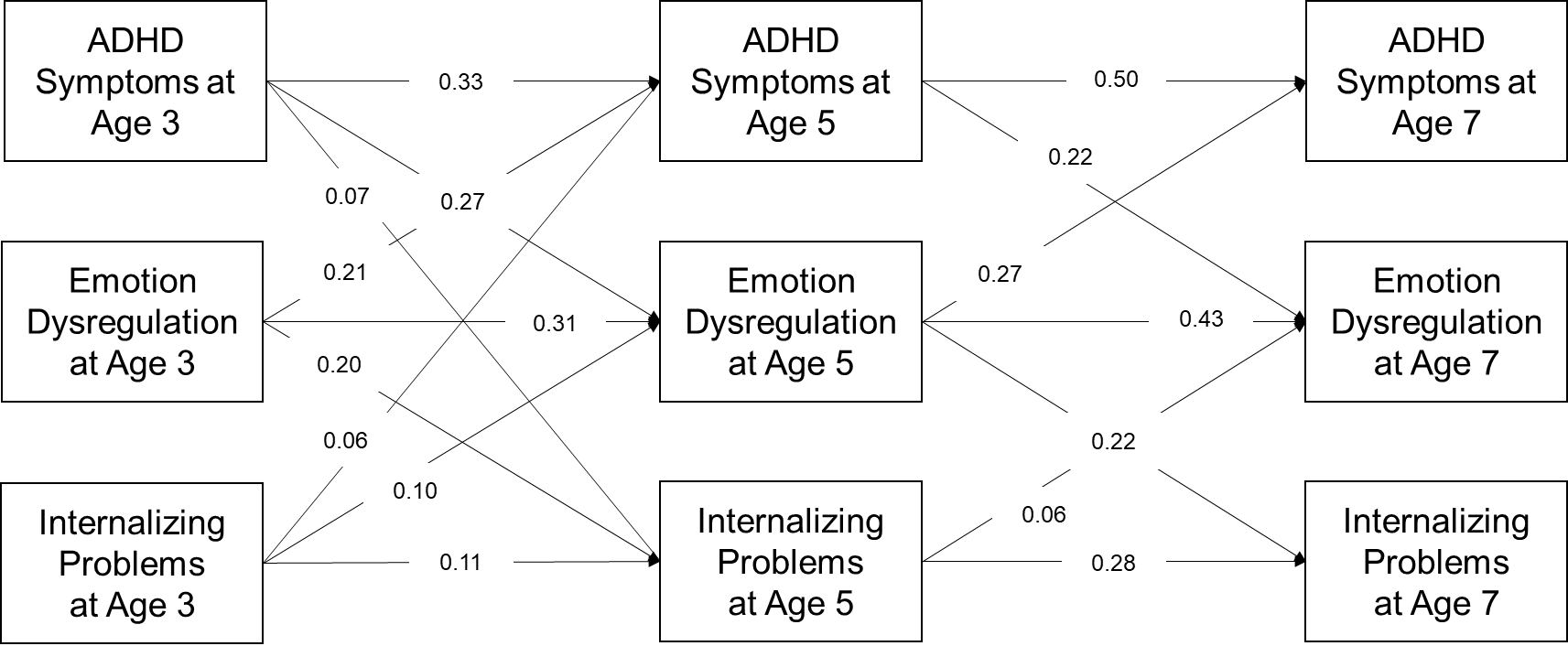
**

**Figure S2: Standardized autoregressive and cross-lagged estimates from the ALT-SR fit to the female subsample**

*Note.* Solid lines represent statistically significant paths at *p* <.05. Latent growth curve and covariance parameters are omitted for clarity.
